# VEXAS syndrome as an underlying cause of disease in patients with Sweet syndrome

**DOI:** 10.1016/j.jdcr.2026.04.015

**Published:** 2026-04-16

**Authors:** Josefien J.L. van Leengoed, Abraham Rutgers, Karina de Leeuw, André B. Mulder, Gilles F.H. Diercks, Marieke C. Bolling, Barbara Horváth, Joost M. Meijer

**Affiliations:** a Department of Dermatology, University of Groningen, University Medical Center Groningen, Groningen, The Netherlands; b Department of Rheumatology and Clinical Immunology, University of Groningen, University Medical Center Groningen, Groningen, The Netherlands; c Department of Laboratory Medicine, University of Groningen, University Medical Center Groningen, Groningen, The Netherlands; d Department of Pathology, University of Groningen, University Medical Center Groningen, Groningen, The Netherlands

**Keywords:** acute febrile neutrophilic dermatosis, autoinflammatory disease, Sweet syndrome, VEXAS syndrome

## Introduction

Dermatological symptoms are the most frequent manifestation of Vacuoles, E1 enzyme, X-linked, Autoinflammatory, Somatic (VEXAS) syndrome^1^ and often precede other manifestations of this severe acquired hemato-inflammatory disorder.^2^ VEXAS syndrome arises from a somatic pathogenic variant in the Ubiquitin-like modifier activating enzyme 1 (UBA1) gene.^1^ As this acquired variant is located on the X-chromosome, the population consists of predominantly male patients aged more than 50 years.^3^ Pathogenic UBA1 gene variants in myeloid precursor cells cause an upregulation of inflammatory pathways, and the consequent systemic inflammation can manifest in a wide range of tissues, leading to a highly variable clinical presentation.^1^^,^^4^ Skin lesions appear in more than 80% of VEXAS patients, including clinical features of Sweet syndrome (acute febrile neutrophilic dermatosis).^4^ Sweet syndrome is a dermatological disorder characterized by fever and tender erythematous papules, plaques, or nodules with a dense dermal neutrophilic infiltrate. The skin lesions may be associated with various, including malignancy, infection and autoimmune or inflammatory disorders, such as VEXAS syndrome.^5^^,^^6^ Notably, Sweet syndrome may present as the first manifestation of VEXAS syndrome.^2^ However, no systematic data are known yet about the prevalence of VEXAS syndrome as underlying cause of Sweet syndrome. Recognizing these lesions as a potential clinical indicator of VEXAS syndrome may aid in timely diagnosis for clinicians, allowing for early and specific treatment. Therefore, this study assessed the prevalence of VEXAS syndrome in a high-risk group of Sweet syndrome patients (male, aged > 50 years).

## Materials and methods

**Fig 1:**
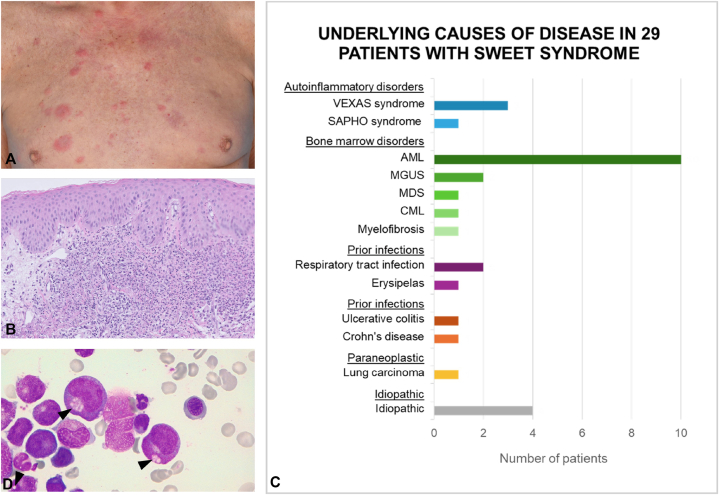
Sweet syndrome and VEXAS syndrome: patient characteristics, clinical, and histopathological features. Sweet syndrome skin lesions in a patient with VEXAS syndrome **(A)**. A skin biopsy with H&E staining showing a dense infiltrate of neutrophils in the dermis, compatible with a diagnosis of Sweet syndrome **(B)**. Underlying causes of disease in 29 patients with Sweet syndrome **(C)**. A bone marrow aspirate with characteristic vacuoles in (myeloid/erythroid) precursor cells, indicated by black arrowheads **(D)**.

This monocenter retrospective cohort study was conducted at the University Medical Center Groningen, a tertiary care hospital with an adherence population of 3 million. High-risk patients for VEXAS syndrome (male, aged > 50 years) were selected from a population of Sweet syndrome patients. This population consisted of 29 patients diagnosed with Sweet syndrome between January 2012 and May 2023. Sweet syndrome was clinically and histopathologically confirmed (Fig 1, *A-C*) for all patients. After selection of high-risk patients, medical records were screened for reported manifestations of VEXAS syndrome, such as autoinflammatory symptoms. Material for genetic analysis was collected from residual bone marrow samples obtained during previous biopsies. Genetic analysis was performed by whole exome sequencing via next generation sequencing. Vacuoles in bone marrow (as shown in Fig 1, *D*) were not assessable retrospectively. One of the VEXAS-positive patients (patient 3 in Table I) was referred to another hospital shortly after the first presentation with skin lesions; data on the presence of inflammatory events are therefore lacking.

**Table I:** Demographical and clinical characteristics of patients with Sweet syndrome with increased risk of VEXAS syndrome

Patient	1	2	3	4	5	6	7	8	9	10
VEXAS syndrome diagnosis	Yes	Yes	Yes	No	No	No	No	No	No	No
Demographics										
Sex, age at SO	M, 69	M, 70	M, 69	M, 77	M, 69	M, 61	M, 53	M, 63	M, 55	M, 69
Status (alive/deceased)	Alive	Deceased	Deceased	Deceased	Deceased	Deceased	Deceased	Deceased	Deceased	Deceased
Follow-up (y)	5	7	7	1, 5	0, 5	1	1	1	0	9
Genetics										
UBA1 variant	p.Met41Leu	p.Met41Leu	p.Met41Leu	Negative	Negative	Negative	Negative	Negative	Negative	Negative
Systemic symptoms										
Fever	No	Yes	-	No	Yes	Yes	Yes	No	Yes	No
Involuntary weight loss	Yes	Yes	-	Yes	Yes	-	Yes	Yes	No	Yes
Night sweats	Yes	No	-	Yes	Yes	Yes	Yes	No	-	No
Malaise	No	Yes	Yes	Yes	Yes	Yes	Yes	Yes	Yes	Yes
Clinical characteristics										
Skin	Sweet syndrome	Sweet syndrome	Sweet syndrome	Sweet syndrome	Sweet syndrome	Sweet syndrome	Sweet syndrome	Sweet syndrome	Sweet syndrome	Sweet syndrome
	SCLE	EAC								
Bone marrow	MDS	ACD	MDS	MDS progressing into AML	AML	MDS progressing into AML	Myelofibrosis progressing into AML	CML	AML	MGUS
	Macrocytic anemia		Macrocytic anemia	Macrocytic anemia	Macrocytic anemia					
Joints and cartilage	Polyarthralgia	Oligoarthritis	Oligoarthritis/Gout	Arthralgia	Arthralgia	-	-	-	-	-
	Arthritis	Relapsing polychondritis								
	Myalgia									
Eyes	Ocular inflammation	Relapsing episcleritis	-	-	-	Periorbital edema	-	-	-	-
Kidneys	TIN	-	-	-	-	-	-	-	-	-
Heart and vascular system	Dilated ascending aorta	DVT abdominal aneurysm	-	-	-	-	Peri-myocarditis	-	-	-
Gastro-intestinal system	Possibly neutrophilic inflammation of the esophagus	-	-	-	-	-	-	-	-	-
Central nervous system	-	Vascular dementia	Cerebral infarction	-	CVA	-	-	-	-	-
Disease progression										
Sweet syndrome related to hematological abnormality	Skin lesions before MDS, but SS 3 months after	Skin lesions before ACD, but SS 2 years after	SS is present 2 years prior to MDS	SS 1 month after MDS/AML diagnosis	SS 1 month after AML diagnosis	SS 1 year after MDS diagnosis	SS 2 months after AML	SS 5 months before presentation with CML	Simultaneous	SS 9 years after MGUS
Cause of death	-	VEXAS and RTI	Unknown	Quick progression of MDS/AML	AML with poor response to chemotherapy	AML and NSTEMI, the latter caused AKI and dyspnea	Multiorgan failure due to septic shock after allo-SCT	CML and end-stage COPD	Extensive complications after chemotherapy for AML	Heart failure and infectious enteritis

*ACD*, Anemia of chronic disease; *AKI*, acute kidney injury; *allo-SCT*, allogeneic stem cell transplantation; *AML*, acute myeloid leukemia; *CML*, chronic myeloid leukemia; *COPD*, chronic obstructive pulmonary disease; *CVA*, cerebrovascular accident; *DVT*, deep venous thrombosis; *EAC*, erythema annulare centrifugum; *M*, male; *MDS*, myelodysplastic syndrome; *MGUS*, monoclonal gammopathy of undetermined significance; *NSTEMI*, non-ST elevation myocardial infarction; *RTI*, respiratory tract infection; *SCLE*, subacute cutaneous lupus erythematosus; *SO*, symptom onset; *SS*, Sweet syndrome; *TIN*, tubulointerstitial nephritis.

## Results

In a population of 29 patients with Sweet syndrome, 10 of 13 male patients were aged more than 50 years, and thus fit the high-risk criteria for VEXAS syndrome (Table I). VEXAS syndrome was confirmed in 3 of 10 high-risk patients (30%). All VEXAS patients carried the p.Met41Leu pathogenic variant of the UBA1 gene.

### Comparative clinical analysis of VEXAS-positive and VEXAS-negative patients

#### Hematological profile

Two of 3 VEXAS-positive patients (67%) had macrocytic anemia at the time of diagnosis, while in the non-VEXAS group, it was observed in only 2 of 7 patients (29%). All high-risk patients were known to have a hematological disorder or malignancy prior to this study (Table I). In the 3 VEXAS-positive patients, hematological disorders consisted of myelodysplastic syndrome (MDS) and anemia of chronic disease (ACD) without progressing disease. In the VEXAS-negative patients, hematological disorders were of a more aggressive nature in 5 of 7 patients (71%) including acute myeloid leukemia or MDS progressing into acute myeloid leukemia.

#### Temporary association

Skin manifestations preceded the diagnosis of MDS or ACD in all 3 VEXAS-positive patients (100%). However, a definitive diagnosis of Sweet syndrome was confirmed after histopathology of a skin biopsy during a later stage of the disease course in 2 of 3 patients. 5 of 7 VEXAS-negative patients (71%) developed Sweet syndrome after the diagnosis of a hematological disease (median: 2 months; range: 1-918 months). Skin lesions were not present prior to diagnosis of a hematological disorder in all 7 VEXAS-negative patients (100%).

#### Systemic inflammation

VEXAS-positive patients demonstrated a wider range of autoinflammatory phenomena with various organ systems affected across patients, mainly consisting of (oligo)arthritis, myalgia, relapsing polychondritis, ocular inflammation/episcleritis, and tubulointerstitial nephritis. In VEXAS-negative patients, systemic symptoms were more often limited to constitutional manifestations.

#### Patient outcomes

At the time of this study, all 7 VEXAS-negative patients (100%) and 2 of 3 VEXAS-positive patients (67%) were deceased. The period between the first presentation of Sweet syndrome and death was longer in VEXAS-positive patients compared to VEXAS-negative patients, with a median survival of 7 years versus 1 year, respectively. The cause of death in one VEXAS-positive patient was disease-related and the other unknown. In 6 of 7 of the VEXAS-negative patients, the cause of death was related to progression of hematological disease or treatment-related adverse events.

## Conclusions

VEXAS syndrome was identified as the underlying cause of disease in 3 of 10 (30%) high risk (males, aged > 50 years) Sweet syndrome patients. All 3 VEXAS-positive patients carried the p.Met41Leu variant, described to be the variant most often associated with Sweet syndrome.^7^ Although the group of high-risk patients with Sweet syndrome was of modest size (*n* = 10), clear distinctions emerged between patients with underlying VEXAS syndrome and those with other hematological disorders. These differences are consistent with current scientific understanding and may aid in determining which Sweet syndrome patients are at risk of having underlying VEXAS syndrome. Key findings include the polysystemic autoinflammatory phenotype, development of cutaneous manifestations before onset of a hematological disorder, and the presence of macrocytic anemia at first presentation. Macrocytic anemia and MDS are described to be the predominant hematological features of VEXAS syndrome and were present in 2 of 3 VEXAS-positive patients in our cohort.^8^ The third was diagnosed with ACD, which has not been reported yet as a manifestation of VEXAS syndrome.

A notable observation was the longer survival following the initial presentation of skin lesions among VEXAS-positive patients, which may be interpreted in several ways. It could suggest a more chronic disease course with inflammation in VEXAS-positive patients, compared with VEXAS-negative patients with more frequently aggressive hematological diseases and treatment adverse events as a cause of death.

Alternatively, patients who develop cutaneous manifestations early on may seek medical attention sooner with early initiation of anti-inflammatory therapy, which may contribute to slowing disease progression and increased survival. Random variation within the small study population may also contribute to this finding. Further investigation is warranted to determine whether these observations persist in a larger prospective study population, which could be challenging due to the low prevalence of Sweet syndrome.^5^

The prevalence observed in this cohort is consistent with the findings of Gurnari et al,^9^ who detected pathogenic *UBA1* variants in 3 of 6 (50%) male patients with Sweet syndrome and a bone marrow disorder. A larger study by Gil-Lianes et al^6^ examined the spectrum of underlying causes of Sweet syndrome in a cohort of 93 patients (2001-2021) and identified 3 patients with confirmed VEXAS syndrome. Taken into consideration the inclusion period, the first report of VEXAS syndrome in 2020, and availability of diagnostic tests, the actual prevalence of VEXAS syndrome may have been underestimated.^1^

Limitations of the study include the modest cohort size and retrospective design, which did not allow us to analyze bone marrow vacuolization, a classic morphological marker. This could lead to an underestimation of the true prevalence if some patients had other undetected mutations of UBA1 or related genes.

VEXAS syndrome was identified in this high-risk cohort of men aged more than 50 years with Sweet syndrome in 3 of 10 patients (30%). Our findings indicate skin lesions of Sweet syndrome could be an early identifier of VEXAS syndrome in this high-risk population. The combination of Sweet syndrome with macrocytic anemia and multisystem autoinflammation should raise suspicion for VEXAS syndrome and is a key indicator for conducting genetic testing for UBA1 pathogenic variants. We recommend that this test be included in the standard diagnostic algorithm to aid early diagnosis, which is critically important for improving prognosis of VEXAS syndrome.

## Conflicts of interest

None disclosed.
